# Well-width dependence of the emission linewidth in ZnO/MgZnO quantum wells

**DOI:** 10.1186/1556-276X-7-605

**Published:** 2012-10-31

**Authors:** Xue-Qin Lv, Jiang-Yong Zhang, Lei-Ying Ying, Wen-Jie Liu, Xiao-Long Hu, Bao-Ping Zhang, Zhi-Ren Qiu, Shigeyuki Kuboya, Kentaro Onabe

**Affiliations:** 1 Pen-Tung Sah Institute of Micro-Nano Science and Technology, Xiamen University, Xiamen, 361005, People's Republic of China; 2 Department of Physics, Xiamen University, Xiamen, 361005, People's Republic of China; 3 State Key Laboratory of Optoelectronic Materials and Technologies, Sun Yat-sen University, Guangzhou, 510275, People's Republic of China; 4 Department of Advanced Materials Science, The University of Tokyo, 5-1-5 Kashiwanoha, Kashiwa, Chiba, 277-8561, Japan; 5 Key Laboratory of Semiconductor Materials Science, Institute of Semiconductors, Chinese Academy of Sciences, P.O. Box 912, Beijing, 100083, People's Republic of China

**Keywords:** ZnO/MgZnO quantum well, photoluminescence, linewidth

## Abstract

Photoluminescence (PL) spectra were measured as a function of well width (*L*_W_) and temperature in ZnO/Mg_0.1_Zn_0.9_O single quantum wells (QWs) with graded thickness. The emission linewidth (full width at half maximum) was extracted from the emission spectra, and its variation as a function of *L*_W_ was studied. The inhomogeneous linewidth obtained at 5 K was found to decrease with increasing *L*_W_ from 1.8 to 3.3 nm due to the reduced potential variation caused by the *L*_W_ fluctuation. Above 3.3 nm, however, the linewidth became larger with increasing *L*_W_, which was explained by the effect related with defect generation due to strain relaxation and exciton expansion in the QW. For the homogenous linewidth broadening, longitudinal optical (LO) phonon scattering and impurity scattering were taken into account. The LO phonon scattering coefficient *Γ*_LO_ and impurity scattering coefficient *Γ*_imp_ were deduced from the temperature dependence of the linewidth of the PL spectra. Evident reduction of *Γ*_LO_ with decreasing *L*_W_ was observed, which was ascribed to the confinement-induced enhancement of the exciton binding energy. Different from *Γ*_LO_, a monotonic increase in *Γ*_imp_ was observed with decreasing *L*_W_, which was attributed to the enhanced penetration of the exciton wave function into the barrier layers.

## Background

ZnO has been attracting much attention recently due to its potential applications in light-emitting devices in the ultraviolet spectral region. An important issue in enhancing the emitting efficiency of optoelectronic devices is the bandgap engineering to form a low-dimensional structure [1-4]. ZnO/MgZnO quantum well (QW) has been considered as one of the most promising structures due to its larger oscillation strength, enhanced binding energy in the excitonic region[1], and tunability of operating wavelength[2]. Up to now, this structure has been demonstrated on various substrates such as ScAlMgO_4_[5], ZnO [6], sapphire [2], and silicon [7]. The optical properties have been investigated widely, including quantum confinement effect [5-7], quantum-confined Stark effect (QCSE) [8-10], temperature dependence of excitonic emission [11-13], localized characteristics of excitons [14-16], and so on. Besides, the linewidth of absorption or photoluminescence (PL) is also crucial to understand the fundamental physics and optical properties of semiconductor microstructure. On the one hand, the structural quality of the QW can be characterized by studying the inhomogeneous broadening generally induced by the well width (*L*_W_) fluctuation and alloy disorder. On the other hand, the value of carrier-scattering parameters in semiconductors, such as longitudinal acoustic phonon, longitudinal optical (LO) phonon, and impurity scatterings, can be extracted from the homogeneous broadening [17,18]. In addition, for optoelectronic device applications such as the laser diode, the linewidth has a direct effect on performance and, especially, is directly related to the lasing threshold. Thus, the linewidth measurement is also of critical importance in the performance of optoelectronic device based on QW. Sun et al. [11] investigated the homogenous linewidth broadening of the excitonic absorption peak in ZnO/MgZnO multi-QWs. Effective reduction of the exciton-LO phonon coupling with decreasing *L*_W_ was observed. However, more detailed study of the dependence of emission linewidth broadening on *L*_W_ was not reported due to the difficulty in sample preparation. In this paper, a special ZnO/Mg_0.1_Zn_0.9_O single QW sample, in which the *L*_W_ was continuously changed from 1.4 to 7.5 nm, was used to evaluate the PL linewidth-broadening mechanisms. It was found that inhomogenous broadening, LO phonon scattering, and impurity scattering contributed to the PL linewidth, and all of them were strongly dependent on the *L*_W_. A detailed analysis of the results was conducted.

## Methods

ZnO/Mg_0.1_Zn_0.9_O single QW was grown by a metalorganic chemical vapor deposition system. Al_2_O_3_ ($11\overset{―}{2}0$) wafers were used as substrates because of the larger critical thickness for ZnO layer-by-layer growth [19]. The sample consists of a three-layer Mg_0.1_Zn_0.9_O/ZnO/Mg_0.1_Zn_0.9_O sandwich structure. The growth temperatures of the Mg_0.1_Zn_0.9_O barrier layer and ZnO well layer were 425°C and 475°C, respectively. By introducing a gradient in the growth rate across the sample, a graded layer thickness was obtained. The details of the growth procedure and method to determine the *L*_W_ can be found elsewhere [2,9]. In order to mark the sample position, a thin film of Au metal was deposited on the sample surface followed by an opening of hole arrays using standard photolithography and liftoff processes [13]. The holes with a diameter of 5 μm were used for the PL measurements, whereas the area without holes was covered with the Au metal. The sample was then characterized by micro-PL spectroscopy from 5 to 300 K. A continuous He-Cd laser operating at 325 nm was used as the excitation source. A reflective objective lens was applied to focus the laser beam to a diameter of approximately 5 μm into the holes. The luminescence from the sample was collected by the same objective lens, dispersed by a spectrometer, and detected using a charge-coupled device. By conducting the laser beam to different holes, the luminescence from different layer thicknesses was obtained.

## Results and discussion

**Figure 1:**
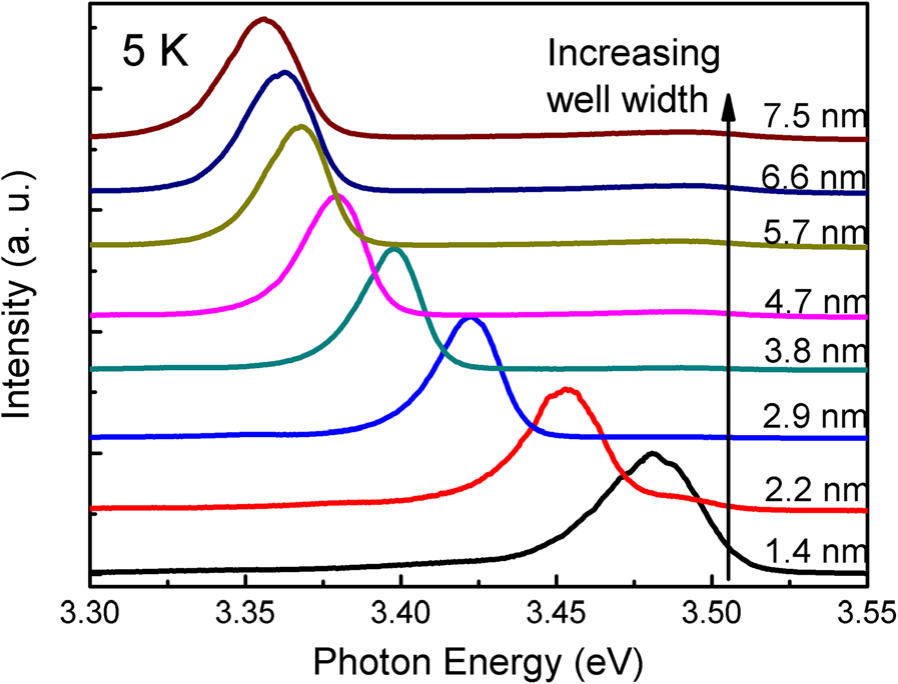
**5**-**K PL spectra of ZnO**/**Mg**_**0.1**_**Zn**_**0.9**_**O single QWs with different well widths from 1.4 to 7.5 nm.** The spectra were normalized and shifted vertically for clarity.

Figure 1 shows the 5-K *L*_W_-dependent PL spectra of the ZnO/Mg_0.1_Zn_0.9_O single QWs. All of the spectra are dominated by a strong peak which was assigned to the radiative recombination of the localized excitons, as described in our previous work [13]. The localization is related to the potential variation induced by the *L*_W_ fluctuation. Obviously, this emission band shifted to higher energy with decreasing *L*_W_. This can be explained by the well-known quantum confinement effect [5-7]. The emission from the barrier layer located at about 3.61 eV was not observed because of the application of a filter with 350-nm (3.54 eV) cutoff wavelength. It should be also noted that the constant PL peak position (3.61 eV) of the Mg_0.1_Zn_0.9_O barrier layers with different layer thicknesses in our previous work is indicative of a negligibly small interdiffusion of chemical species at the ZnO/MgZnO interfaces [13].

**Figure 2:**
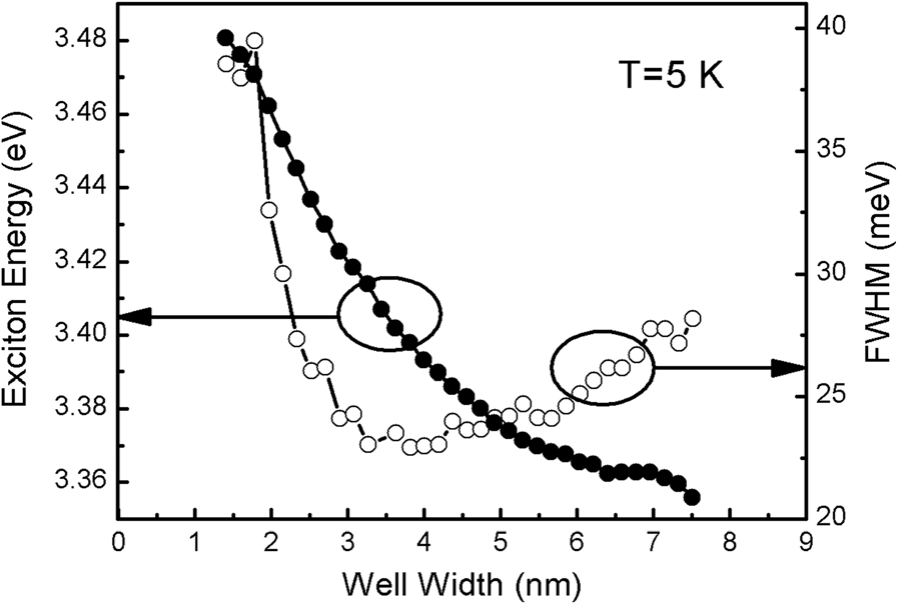
**Peak energy and FWHM of exciton emission spectra as a function of well width.** The solid and open circles represent the peak energy and FWHM of the exciton emission, respectively.

The peak energy and full width at half maximum (FWHM) of the excitonic emission spectra are given as a function of *L*_W_ in Figure 2, which were extracted from the emission spectra in Figure 1. It is clear that as the *L*_W_ increases from 1.4 to 7.5 nm, the emission energy decreases from 3.481 to 3.356 eV. The reason has been explored in our previous work by comparing the measured and calculated QW PL energy [13]. For *L*_W_ bellow 3 nm, the reduction of the exciton energy with increasing *L*_W_ can be attributed to the weakening of quantum confinement effect. While above 3 nm, the QCSE induces a spatial separation of electrons and holes leading to a further redshift of the PL energy in QW. A good agreement between the measured and calculated PL peak position for the well layer below 3 nm further confirms the negligible interdiffusion of chemical species at the ZnO/MgZnO interfaces. However, the FWHM as a function of *L*_W_ behaves differently from peak energy. The FWHM is found to decrease from 1.8 to 3.3 nm and then increase monotonically with increasing *L*_W_. In the small-*L*_W_ range, quantum confinement effect is the dominant mechanism. It is known that the confinement potential is sensitive to the *L*_W_, and the same *L*_W_ variation may induce larger potential fluctuation in the narrower well so that in this region the FWHM represents an inhomogeneous linewidth broadening mainly induced by *L*_W_ fluctuation. Furthermore, as the well thickness increases, the strain in the QW caused by the lattice mismatch between ZnO and Mg_0.1_Zn_0.9_O would be relaxed to reduce the accumulated strain energy by generating additional defects. Consequently, a quenching of exciton emission and linewidth broadening occur. Besides, the internal electric field induced by spontaneous and piezoelectric polarizations may play a significant role [20]. It is known that the electrons and holes are separated by a distance along the growth axis by the internal electric field. Therefore, the excitons expand in the QW and can be captured by defects more easily. It is thus easy to infer that the generated defects and spatial redistribution of the electrons and holes must be responsible for the observed increase of FWHM in the large-*L*_W_ range.

**Figure 3:**
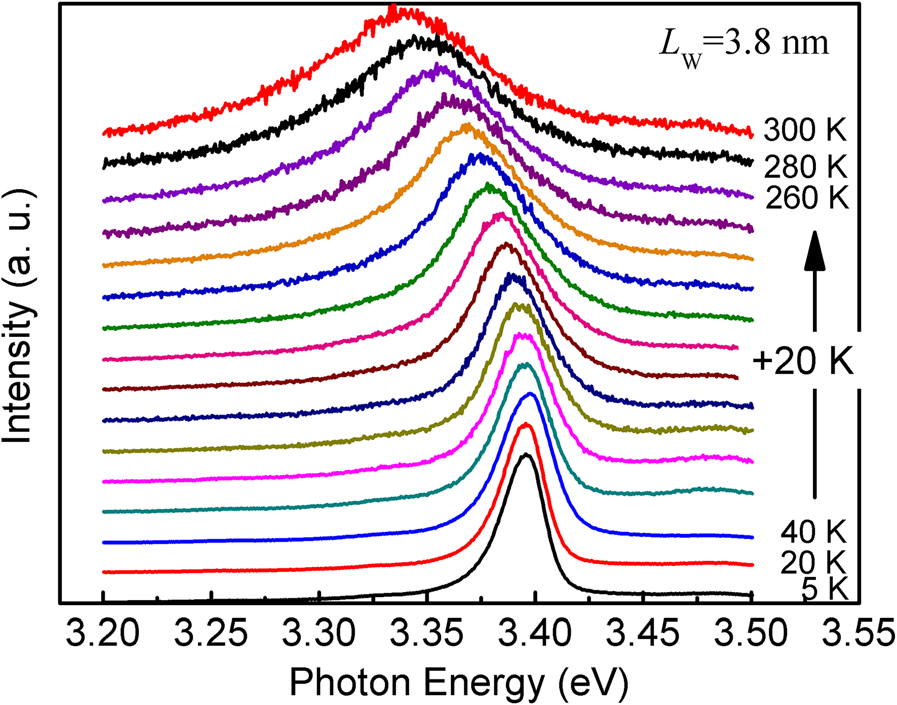
**ZnO**/**Mg**_**0.1**_**Zn**_**0.9**_**O single QW PL spectra with a well width of 3.8 nm at various temperatures.** The spectra were normalized and shifted vertically for clarity.

**Figure 4:**
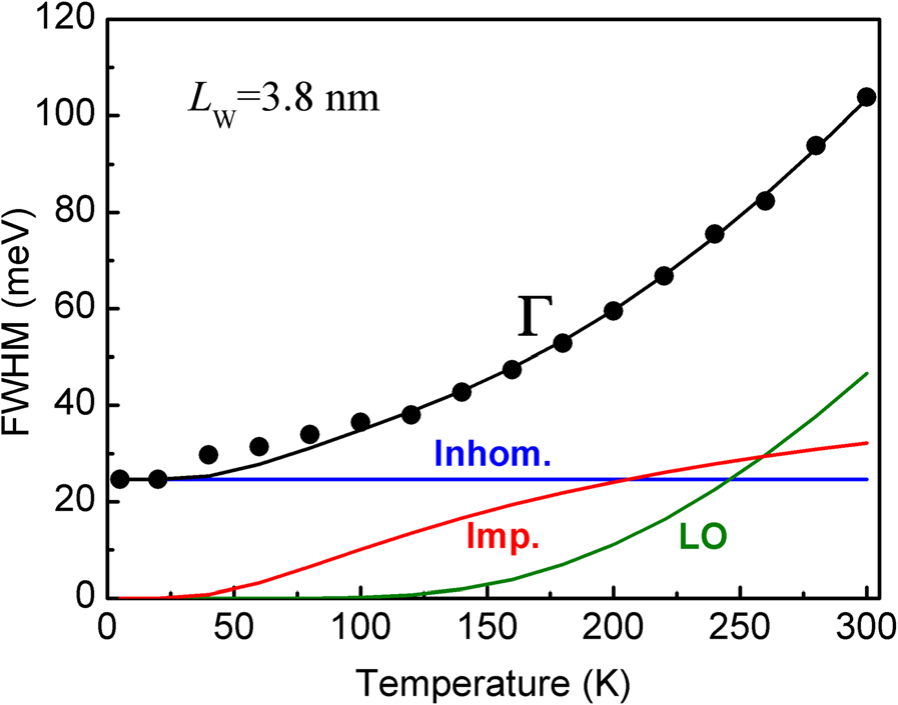
**Temperature dependence of experimental and fitted results of FWHM.** The solid circles indicate the experimental FWHM extracted from the emission spectra in Figure 3. The solid black line represents the fit according to Equation 1. The colored lines represent the contributions to the FWHM from the inhomogeneous linewidth broadening (Inhom.) and interactions with LO phonons (LO) and impurities (Imp.).

In order to analyze the homogenous broadening mechanism, temperature-dependent PL measurement was carried out. Figure 3 shows the PL spectra of a typical QW with a *L*_W_ of 3.8 nm from 5 to 300 K. The extracted FWHM from the emission spectra in Figure 3 is given as a function of temperature in Figure 4 (solid circles). In agreement with the results reported by Sun et al. [11] and Misra et al. [21], the FWHM grows sublinearly as the temperature increases from 5 to 180 K but rises more sharply as the temperature is raised higher than 180 K. In order to interpret this type of temperature dependence, the experimental FWHM data were fitted using a model that includes three types of broadening mechanisms [17,18]:

(1) $$\begin{array}{l} \Gamma \left(T\right)\;=\;\Gamma_{\text{inh}}\;+\;\frac{\Gamma_{\text{LO}}}{\exp\left(\hbar \omega_{\text{LO}}/k_{\text{B}}T\right)-1}\\ \;+\;{\text{Γ}}_{\text{imp}}\exp\left(-\frac{E_{\text{B}}}{k_{\text{B}}T}\right) \end{array}$$

where *Γ*_inh_ is the inhomogenous broadening due to the fluctuation of well thickness. *Γ*_LO_ is the LO phonon scattering coefficient (with a LO phonon energy *ћω*_LO_ = 72 meV), *Γ*_imp_ is the impurity scattering coefficient, and *E*_B_ is the average binding energy of the impurity-exciton complexes. The solid black line in Figure 4 represents the fitted result based on Equation 1. The best fit was obtained for the parameter values *Γ*_inh_ = 24.6 meV, *Γ*_LO_ = 708 meV, *Γ*_imp_ = 57 meV, and *E*_B_ = 15 meV. It should be noted that the extracted average binding energy of the impurity-exciton complexes coincides with the donor-exciton localization energy in ZnO [22,23]. Therefore, we speculate that the donors, such as oxygen vacancies [24], zinc interstitials [24], hydrogen [25], etc induced by unintentional n-doping, are the main defects or impurities which broaden the exciton emission spectra.

The individual contributions to the FWHM from inhomogenous broadening and interactions with LO phonon and impurities are presented with colored lines in Figure 4. It is seen that except for inhomogenous broadening, below 250 K, the impurity scattering mainly contributes to the FWHM. As the temperature increases above 250 K, scattering by LO phonons becomes the main temperature-dependent contributor due to the increasing LO phonon population.

**Figure 5:**
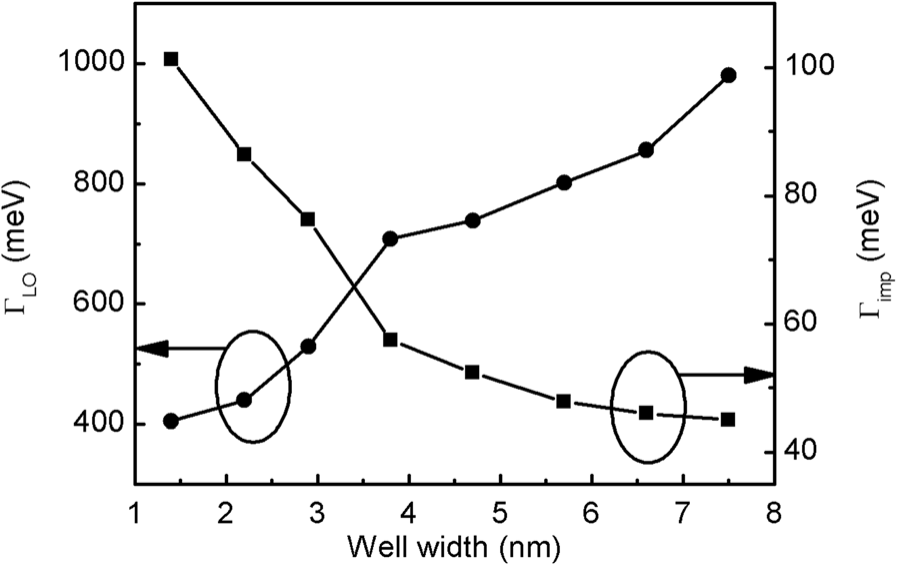
**LO phonon scattering coefficient*Γ***_**LO**_**and impurity scattering coefficient*Γ***_**imp**_**as a function of well width.** The solid circles and squares represent *Γ*_LO_ and *Γ*_imp_, respectively.

We made the same fitting procedure for other QWs and summarized the obtained values of *Γ*_LO_ and *Γ*_imp_ for different *L*_W_s in Figure 5. It can be seen that there is a monotonic decrease in the *Γ*_LO_ as the *L*_W_ is reduced. This result is related to the LO phonon and exciton scattering process via the Fröhlich interaction [26]. We know that 1*s* exciton either is totally ionized into the free electron–hole continuum or scatters within the discrete exciton bands by absorbing one LO phonon with the energy *ћω*_LO_. This process contributes to the exciton linewidth broadening, while the dissociation channel of the excitons into the continuum state by 1-LO phonon absorption is inhibited when the exciton binding energy is larger than the phonon energy. Nevertheless, the transition from 1*s* to other excited exciton states (such as the 2*s* and 2*p* states) is still possible. The exciton binding energy of bulk ZnO is 60 meV, and the LO phonon energy is 72 meV. However, for the ZnO/Mg_0.1_Zn_0.9_O QW, the exciton binding energy will be enhanced as the *L*_W_ is reduced due to the quantum confinement effect [1]. Therefore, we interpret the strong reduction in the *Γ*_LO_ as being a manifestation of the fact that with the decrease of *L*_W_, the exciton binding energy is enhanced gradually. A similar result has been reported by Sun et al. [11].

On the other hand, different from *Γ*_LO_, a monotonic increase in *Γ*_imp_ is observed with decreasing *L*_W_. The coefficient *Γ*_imp_ is thought to be a measurement for the scattering of shallow donor defects and impurities such as oxygen vacancies, zinc interstitials, hydrogen, etc. Generally, the *Γ*_imp_ depends on the density of defect and impurity sites. Contrary to the distribution of defects in the ZnO QW with different *L*_W_s as indicated in the above analysis, we suppose that defects, impurities, and composition fluctuation in the barrier layers are the dominant scatters. For the narrow QW geometry, the exciton wave function penetrates deeply into the adjacent barrier layers [27], and therefore, the scattering coming from the barrier layers is remarkable. As the well thickness increases, the extension of the exciton wave function into the barrier layers is suppressed; hence, the influence of defect and impurity scattering was sufficiently reduced, leading to a decrease of *Γ*_imp_ with increasing *L*_W_. In addition, in the large-*L*_W_ range, the defects induced by the strain relaxation in the QW may also contribute to the scattering process, showing a slow decreasing trend in *Γ*_imp_ with increasing *L*_W_.

## Conclusions

In conclusion, the broadening mechanisms of the PL excitonic linewidth were investigated in ZnO/Mg_0.1_Zn_0.9_O single QWs with graded thickness. The in homogenous broadening obtained from the 5-K *L*_W_-dependent PL spectra decreased first and then increased with increasing *L*_W_. This was mainly explained by the reduced potential fluctuation and the generated defects in the QW by strain relaxation, respectively. Furthermore, the homogenous broadening mechanisms including LO phonon scattering and impurity scattering were determined by fitting the temperature-dependent PL linewidth to a theoretical model. The LO phonon scattering coefficient *Γ*_LO_ and impurity scattering coefficient *Γ*_imp_ showed different *L*_W_ dependence. The monotonic decrease in *Γ*_LO_ with decreasing *L*_W_ was explained in terms of the confinement-induced enhancement of the exciton binding energy, while the continuous increase in *Γ*_imp_ with decreasing *L*_W_ was attributed to the enhanced penetration of the exciton wave function into the barrier layers.

## Competing interests

The authors declare that they have no competing interests.

## Authors' contributions

The work presented here was carried out in collaboration among all authors. XQL and BPZ designed the study. XQL performed the research and prepared the manuscript. BPZ carried out the experiments and analyzed the data. JYZ, LYY, WJL, and XLH analyzed the data and discussed the analysis. ZRQ, SK, and KO participated in the experiments. All authors read and approved the final manuscript.
